# Comparing bone tissue engineering efficacy of HDPSCs, HBMSCs on 3D biomimetic ABM-P-15 scaffolds in vitro and in vivo

**DOI:** 10.1007/s10616-020-00414-7

**Published:** 2020-08-20

**Authors:** Yamuna Mohanram, Jingying Zhang, Eleftherios Tsiridis, Xuebin B. Yang

**Affiliations:** 1 Biomaterials & Tissue Engineering Group, Department of Oral Biology, School of Dentistry, University of Leeds, Level 7, Wellcome Trust Brenner Building, St. James’s University Hospital, Leeds, LS9 7TF UK; 2 The Second Clinical Medical College, Guangdong Medical University, Dongguan, 523808 Guangdong China; 3 Academic Orthopaedic Department, Aristotle University Medical School, 54124 Thessaloniki, Greece

**Keywords:** PepGen P-15, HDPSCs, HBMSCs, Bone tissue engineering, In vivo

## Abstract

Human bone marrow mesenchymal stem cells (HBMSCs) has been the gold standard for bone regeneration. However, the low proliferation rate and long doubling time limited its clinical applications. This study aims to compare the bone tissue engineering efficacy of human dental pulp stem cells (HDPSCs) with HBMSCs in 2D, and 3D anorganic bone mineral (ABM) coated with a biomimetic collagen peptide (ABM-P-15) for improving bone-forming speed and efficacy in vitro and in vivo. The multipotential of both HDPSCs and HBMSCs have been compared in vitro. The bone formation of HDPSCs on ABM-P-15 was tested using in vivo model. The osteogenic potential of the cells was confirmed by alkaline phosphatase (ALP) and immunohistological staining for osteogenic markers. Enhanced ALP, collagen, lipid droplet, or glycosaminoglycans production were visible in HDPSCs and HBMSCs after osteogenic, adipogenic and chondrogenic induction. HDPSC showed stronger ALP staining compared to HBMSCs. Confocal images showed more viable HDPSCs on both ABM-P-15 and ABM scaffolds compared to HBMSCs on similar scaffolds. ABM-P-15 enhanced cell attachment/spreading/bridging formation on ABM-P-15 scaffolds and significantly increased quantitative ALP specific activities of the HDPSCs and HBMSCs. After 8 weeks in vivo implantation in diffusion chamber model, the HDPSCs on ABM-P-15 scaffolds showed extensive high organised collagenous matrix formation that was positive for COL-I and OCN compared to ABM alone. In conclusion, the HDPSCs have a higher proliferation rate and better osteogenic capacity, which indicated the potential of combining HDPSCs with ABM-P-15 scaffolds for improving bone regeneration speed and efficacy.

## Introduction

The increasing clinical demand for bone regeneration and repair in the context of our ageing population poses a challenge both to healthcare providers and society (Iaquinta et al. 2019). There is also increasing demand for the implant osseointegration, which is crucial for successful implantology in both orthopaedics and dentistry (Chandran and John 2019; Liu et al. 2019). Tissue engineering provides a promising strategy to meet this clinical demand by developing functional bone construct using stem/stromal cells, biomimetic biomaterial scaffolds, with/without growth factors (Abdulghani and Mitchell 2019). However, the main challenge is to identify the most appropriate combination of the three elements that can be used to achieve optimum regeneration of damaged bone tissue (Panetta et al. 2009).

Under in vitro conditions, mesenchymal stem cells (MSCs) exhibit the ability to form fibroblastic colonies on tissue culture plastic (Gothard et al. 2013) and can differentiate alone osteoblast, chondrocyte, adipocytes, and other different lineages when cultured under the appreciate inductive media (Garcia-Sanchez et al. 2019). HBMSCs has been considered as one of the most popular stem cell sources for stem cells therapy and bone tissue engineering (Connolly et al. 1989; Kern et al. 2006; Squillaro et al. 2016; Yoshii et al. 2009). However, bone marrow biopsy/aspiration itself is an invasive procedure, and in elderly patients, they often lack good quality and quantity of desired stem cells within the bone marrow (Yamada et al. 2014). It has been documented that the poor response of these cells is due to the loss of potential to proliferate and differentiate with increasing donor age (Jones and Schafer 2015; Kern et al. 2006; Muschler et al. 2001; Yamada et al. 2010; Yoshii et al. 2009). Taken together, these factors have led to the search for an alternative adult stem cell sources which can be easily accessed with minimal invasion and provide the stem cells with similar or better regenerative potential as HBMSCs. In nature, every individual, during their lifetime, experiences teeth loss (80% of subjects had lost one or more tooth, and the mean tooth loss was 5.09)(Ribeiro et al. 2015), which provides an opportunity to access dental tissues with minimal invasion making the option of isolating of stem cells from dental pulp a promising alternative source to HBMSCs. Pulp tissues can be obtained from either permanent or deciduous teeth, however, wisdom teeth (third molars) have long been a preferred choice of the permanent teeth (Ledesma-Martinez et al. 2016). This may due to the third molars are routinely extracted due to impaction caused by the lack of jaw space, and it is also the last permanent teeth to erupt, and their pulp tissue is considered to be rich in unspecialised cells (Gronthos et al. 2000; Ledesma-Martinez et al. 2016). A number of studies showed that HDPSCs is a small population of cells residing in the pulp tissue which exhibits a highly proliferative and multi-lineage differentiation ability (Cui et al. 2014; Gronthos et al. 2000; Mortada and Mortada 2018). These cells are thought to play a role in the repair of damaged pulp and dentine by differentiating into specialised cells—odontoblasts secreting dentine matrix. Extensive research has since been carried out pursuant to a good understanding of HDPSCs and their potential in tissue engineering (Kawashima and Okiji 2016).

In natural conditions, type I collagen is predominantly present in the bone extracellular matrix. It not only provides the substrate for cell attachment and migration but also influences the osteogenic differentiation of the adhered cells. Thus, there has been an increasing interest in the application of type I collagen for bone tissue engineering (Weisgerber et al. 2016). Structurally, individual type I collagen molecules are triple helical structures, comprising of two α1 and one α2 polypeptide chains. Each of these chains contains approximately 1000 amino acid residues and is twisted into a right-handed helix. A number of studies have shown that the exposed half turns of the helical structure act as cell-binding sites, through which collagen interacts with cell surface integrin receptors (Murray et al. 2003; Rodwell and Kennelly 2000; Xu et al. 2000). As a result, collagen triggers the signalling pathway to direct the cells in attachment, migration and osteogenic differentiation (Bhatnagar et al. 1999b; Emsley et al. 2000). A synthetic analogue of this cell-binding domain was produced synthetically to mimic the function of the collagen molecule under in vitro conditions for osteogenic induction in cells. This synthetic protein is referred to as “peptide 15” or “P-15” (Bhatnagar et al. 1999b, 1997; Scaria et al. 1989). The function of P-15 on its own has been tested on osteoblastic cell lines—MG63 and HBMSCs (Carinci et al. 2004; Sollazzo et al. 2009). Based on microarray analysis, osteoblastic cells were observed to up-regulate fibronectin, cell cycle and signal transduction related genes after culture in P-15 (Carinci et al. 2004). P-15 peptide under in vitro conditions was observed to function similar to the collagen by influencing the up-regulation of bone-specific proteins in HBMSCs.

In the case of bone regeneration, it is anticipated that an ideal bone graft substitute provides all the essential features of an autologous bone graft, including both the organic and inorganic components of the natural bone. With this concept in mind, a three-dimensional scaffold material was designed by incorporating P-15 peptides on ABM particles, a natural xenogenic source of hydroxyapatite (HA) (Bhatnagar et al. 1999b). These bovine bone chips are pre-treated at high temperatures to remove the organic components of the bone, leaving only the inorganic components (Bhatnagar et al. 1999b; Hofmann et al. 2007; Yuan et al. 2007), which is the major inorganic constituent of natural bone (Neshati et al. 2012). ABM-P-15 mimics the structural framework of the autologous bone graft by supplying both the cell-binding domain of type I collagen and HA for the growth of the cells. To date, ABM-P-15 scaffolds have been successfully tested on both animal models and on humans (Emecen et al. 2009), which demonstrated that P-15 adsorbed on ABM scaffolds enhanced attachment, growth and osteogenic differentiation of the tested cells when compared with ABM scaffolds alone. By far, extensive work has been carried out on the application of ABM-P-15 scaffolds on its own and using different cell types for bone tissue engineering application (Barboza et al. 2002; Lindley et al. 2010; Mardas et al. 2008; Matos et al. 2011; Sarahrudi et al. 2008; Scarano et al. 2003; Thorwarth et al. 2005; Vastardis et al. 2005; Yang et al. 2004). The aim of this study was to compare the osteogenic potential of HDPSCs with HBMSCs and the effect of P-15 on the bone-forming capacity of HDPSCs in vitro and in vivo for the potential of combining these two to improve the bone regeneration efficacy in the clinical setting.

## Materials and methods

Tissue culture reagents were obtained from Corning Life Sciences B.V. (The Netherlands). Alpha-modified minimal essential media (α-MEM) without l-glutamine was purchased from Lonza (UK) and fetal bovine serum (FBS) was from Biosera (UK). Molecular biology reagents were purchased from Invitrogen (UK). Dexamethasone, alkaline phosphatase kits, and all other biochemical reagents were of analytical grade from Sigma (UK) unless otherwise stated.

### Scaffold synthesis and preparation

Two types of scaffolds were used in this study: anorganic bovine mineral (Osteo-Graf/N-300) absorbed with/without P-15, which are FDA approved for the dental application and are commercially available as PepGen P-15^®^ (Cerapedics, Inc. CO, USA). The particles are described in our previous paper (Yang et al. 2004). The 48 well tissue culture plates were coated with 12% poly(2-hydroxyethyl methacrylate)(Poly Sciences, PA) to prevent cell attachment to the plastic. 35 mg of ABM-P-15 or ABM particles were transferred into the well and sterilised using UV radiations for 30 min.

### Isolation and culture of HDPSCs and HBMSCs

Sound third molar teeth were extracted at Leeds School of Dentistry with patients’ informed consent and ethical approval (LREC 07/H1306/93). A total of 20 human teeth was collected (average age: 24 ± 4 years). HDPSCs were isolated and in vitro expanded as previously described (El-Gendy et al. 2013, 2015; Gronthos et al. 2000; Ricordi et al. 1992). 4 human bone marrow samples (average age: 59 ± 16 years) were obtained from routine total hip replacement patients at Leeds General Infirmary and Chapel Allerton Hospital with patients’ informed consent ethical approval by the NHS local ethical committee (COREC: 06/Q1206/165). HBMSCs were isolated and in vitro expanded as previously described (Yang et al. 2001). HDPSCs and HBMSCs were seeded at 2 × 10^5^ cells/well on 35 mg ABM-P-15 and/or ABM particles and were cultured in 500 μL of basal media (α-MEM supplemented with 10% FBS, 1% penicillin/streptomycin, 2 mM l-glutamine) in an incubator (Binder, Germany) at 37 °C with 5% CO_2_.

### Multi-lineage inductive culture of HDPSCs and HBMSCs

For osteogenic culture, HDPSCs and HBMSCs were seeded in 24 well plates (2 × 10^4^ cells/well, P3, n = 3) and cultured for 3 weeks at 37 °C, 5% CO_2_ in osteogenic media (basal media supplemented with 10 nM dexamethasone and 100 μM l-ascorbic acid 2-phosphate). Basal medium alone was used as the controls for both cell groups. The media were changed every 5 days until the cells were harvested for alkaline phosphatase staining.

For adipogenic culture, hDPSCs were seeded onto 24 well plates (2 × 10^4^ cells/well, P3, n = 3) and cultured for 3 weeks at 37 °C and 5% CO_2_ in adipogenic induction media—basal medium supplemented with 1 µM dexamethasone (Sigma), 200 µM indomethacin, 0.5 mM isobutyl-methyl xanthine (Sigma) and 10 µg/mL insulin. The basal medium alone was used as the control. The cells were fixed in 10% neutral buffered formalin (NBF) and were then stained with 0.6% Oil red O (Sigma) for 15 min for the identification of lipid droplets.

For chondrogenic culture, HDPSCs and HBMSCs (5 × 10^5^ cells/mL; P3; n = 3) were cultured as pellets in the basal media for 48 h before transferring into chondroinductive media and maintained at 37 °C, 5% CO_2_ for 3 weeks with media changes every 3 days. Basal medium alone was used as the control group.

The chondroinductive medium was prepared by supplementing the basal media with 0.1 μM dexamethasone (Sigma, UK), 10 ng/mL TGF β3, 50 μg/mL l-ascorbic acid 2-phosphate (Sigma, UK) and 5 μg/mL insulin transferrin selenium (ITS) (Sigma, UK). All cell pellets were, paraffin-embedded, sectioned and stained with Alcian blue/Sirius red for the detection of GAG and collagenous matrix. HDPSCs/HBMSCs growth on the ABM-P-15 and/or ABM scaffold materials was investigated using a confocal microscope, where a series of X–Y–Z images were taken through the scaffold particles permitting 3D reconstruction.

### Assessment of cells viability and growth on ABM-P-15 and ABM scaffolds

At different time points (24 h, 14 days and 6 weeks), HDPSCs and HBMSCs cultured on ABM-P-15 and ABM particles were fluorescently labeled with CellTracker™ Green (CMFDA). Viable cells were imaged under an inverted fluorescent microscope or the Leica confocal microscope (AOBS, UK).

### Scanning electron microscopy

After 6 weeks culturing of HDPSCs and HBMSCs on ABM-P-15 and/or ABM scaffolds, the samples were vacuum dried for 16 h and sputter-coated with gold using an E5000 sputter coater (Polaron, UK) to a thickness of 20 nm prior being imaged under a Hitachi S-3400 N/Nx scanning electron microscope (Hitachi High Technologies, Japan).

### Alkaline phosphatase staining

After fixation in 98% ethanol, the scaffold constructs were incubated in a solution containing 400 μL 0.25% Naphthol AS-MX phosphate (Sigma, UK), 2.4 mg of Fast Violet salt in 10 mL distilled water at 37 °C for 30 min (in darkness). Cells expressing alkaline phosphatase enzymes were stained in red colour.

### Alkaline phosphatase specific activity (ALPSA) quantification

ALP was quantified in HDPSCs and HBMSCs cultured either as monolayers or on 3D ABM-P-15 and/or ABM scaffolds as described previously (Lu et al. 2014; Yang et al. 2003) using a fluorescence spectrophotometer (Fluoroskan ascent, Thermo UK) at 520 nm. Then the ALP activities were normalised to the relevant total DNA content to get the ALPSAs. Statistical analysis was carried out using one-way analysis of variance test with Tukey–Kramer multiple comparisons test. The software used for statistical comparison was GraphPad Instant Software (GraphPad Software, Inc., SanDiego).

### In vivo implantations

Previously, we have reported that ABM-P-15 enhanced HBMSCs bone formation in vivo compared to the ABM scaffold alone (Yang et al. 2004). In this study, we investigated the osteogenic capacity of HDPSCs on ABM-P-15 particles to explore its potential for bone tissue engineering under the Home Office project license (40/2953). Briefly, HDPSCs (130 mL containing 5 × 10^6^ cells per chamber) were injected into diffusion chambers (Millipore, Bedford, MA) containing ABM-P-15 or ABM alone (n = 4), which were implanted intraperitoneally in MF1 Nu/Nu mice as previously described (Lu et al. 2014) for up to 8 weeks.

### Alcian blue/Sirius red staining

The samples were partially demineralised in 10% EDTA (pH 7.4) for 2 weeks and embedded in paraffin. The sections were stained with Alcian blue (0.5 g in 1% acetic acid in water; Sigma, UK) for 10 min and then immersed in 1% aqueous phosphomolybdic acid (Fluka, UK) prior to being stained with 0.3% picrosirius red (Fluka, UK) for an hour.

### Immunofluorescence staining

The sections were firstly incubated in primary antibodies including COL1 (1/50, overnight), OCN (1/50, 1 h) and OPN (1/100) which were followed by incubation for 1 h in FITC-labelled secondary antibodies (goat anti-mouse for COLI, and/or swine anti-rabbit for OCN). The omit of primary antibody was used as the negative control. The sections were then washed in 1 × PBS with agitation for 2 h and the nuclei stained with TO-PRO-3^®^ at 1/100 in PBS for 20 min. The images were taken under a confocal microscope.

## Results

### Multi-lineage differentiation capacity of HDPSCs compared to HBMSCs in monolayer culture

**Fig. 1:**
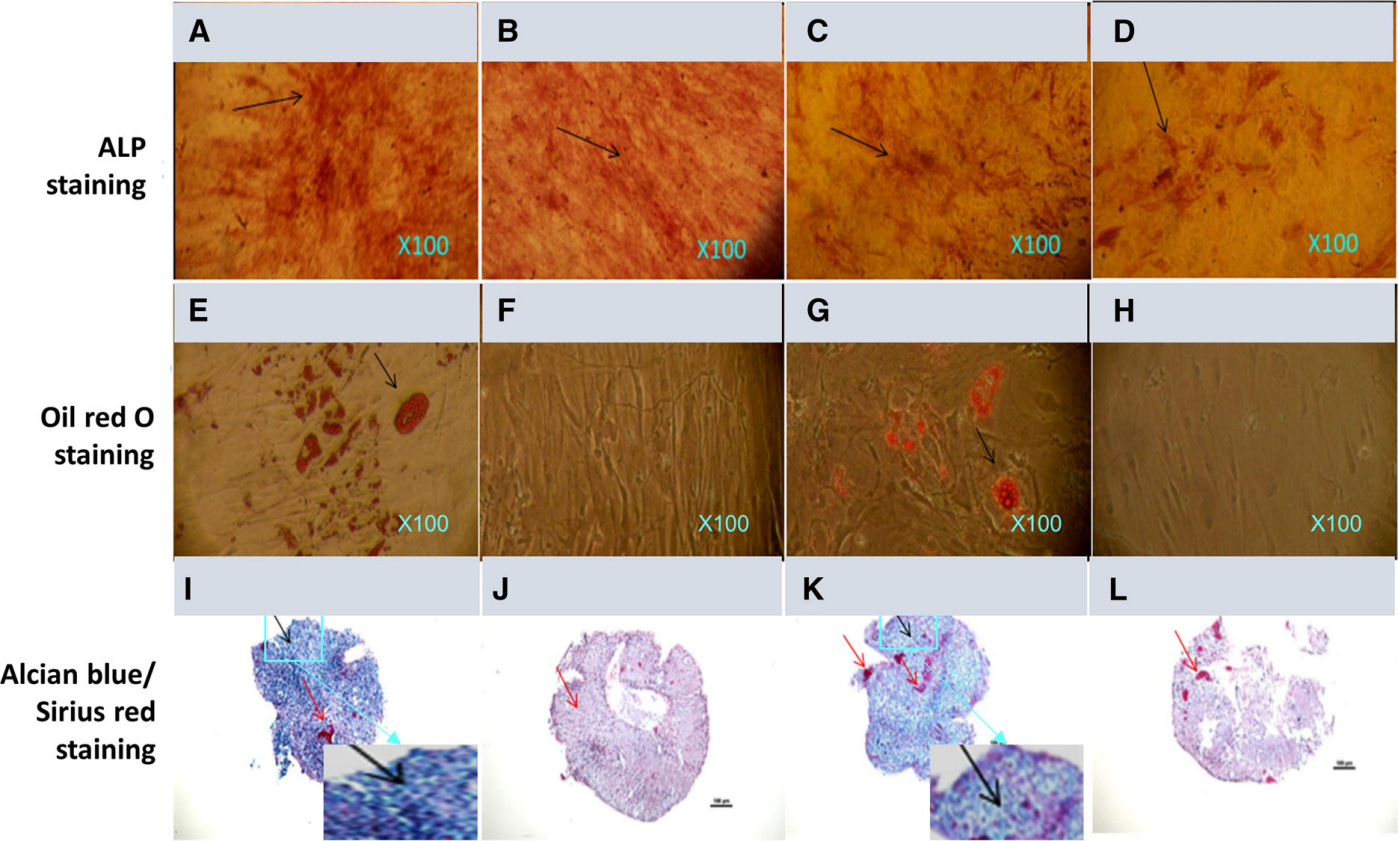
Histological staining of HDPSCs (**a**, **b**, **e**, **f**, **i**, **j**) and HBMSCs (**c**, **d**, **g**, **h**, **k**, **l**) after 3 weeks of culture under osteogenic (**a**, **c**), adipogenic (**e**, **g**), chondrogenic (**i**, **k**) inductions and basal conditions (**b**, **d**, **f**, **h**, **j**, **l**). **a**–**d** ALP staining; **e**–**h** Oil red O staining; **i**–**l**) Alcian Blue/Sirius red staining. Scale bars-100 μm

After 3 weeks of culture, HDPSCs showed much stronger ALP positive staining (red colours. Black arrows) in both osteogenic conditions (Fig. 1a), and basal medium (Fig. 1b) compared to that of HBMSCs in the same culture conditions (Fig. 1c & d) respectively. Osteogenic inductive culture enhanced the ALP staining in both cell groups compared to the same cells in the basal medium culture. After 3 weeks of culture in adipogenic inductive media, Oil red O staining showed that adipogenic culture condition induced lipid droplet formation in both HDPSCs (Fig. 1e) and HBMSCs (Fig. 1g) groups compare to the same cells in basal medium culture condition (Fig. 1f, h) respectively. However, there was no notable difference in staining between HDPSCs and HBMSCs. After 3 weeks of pellet culture in chondrogenic media, both HDPSCs (Fig. 1i) and HBMSCs (Fig. 1k) samples were stained strongly positive for Alcian blue staining probably reflecting sulphate glycosaminoglycans (GAGs: blue colours) with the sparse presence of collagen (red colours) which was indicated when the pellets were stained up by Sirius red (red colour). There were some chondrocyte-like cells within the pellets and somewhere, the chondrocyte-like cells aligned in column-oriented in certain directions (Fig. 1i, k and the inserts: black arrows). In comparison, both cells in the basal medium culture condition appeared to lack of blue staining (Fig. 1j and l).

### HDPSCs/HBMSCs viability and spreading on ABM-P-15 and ABM scaffolds

**Fig. 2:**
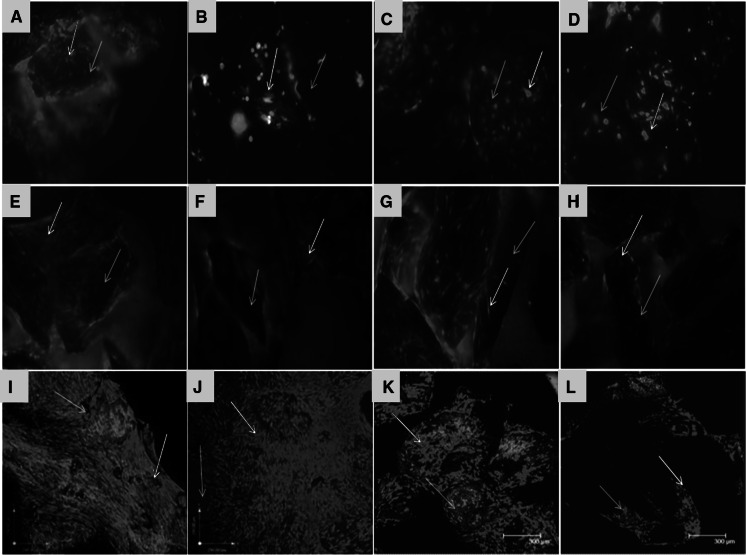
Fluorescent micrographs from an inverted fluorescent microscope (**a**–**h**) and confocal microscope (**i**–**l**) of CMFDA labelled HDPSCs (**a**, **b**, **e**, **f**, **i**, **j**) and HBMSCs (**c**, **d**, **g**, **h**, **k**, **l**) after 24 h (**a**–**d**), 4 days (**e**–**h**) and 6 weeks (**i**–**l**) of in vitro cultures on ABM-P-15 (**a**, **e**, **i**, **c**, **g**, **k**) and ABM scaffolds (**b**, **f**, **j**, **d**, **h**, **l**) (n = 3). Red arrows: viable cells; blue arrows: ABM-P-15/ABM particle. Magnifications: ×100

After 24 h of cell seeding (n = 3), CMFDA fluorescent labelling showed that the majority of both cells on ABM-P-15 and ABM alone are viable. HDPSCs (Fig. 2a) and HBMSCs (Fig. 2c) were observed to have more cell attachment and better spreading on the scaffolds in ABM-P-15 groups in comparison to that of the ABM alone group (Fig. 2b and d), where the most of the particles only have a few cells attached. After 14 days of culture in basal media, HDPSC showed better cell spreading, and proliferation (cell density), cell bridging formation on ABM-P-15 (Fig. 2e) compare to HBMSCs on the ABM-P-15 scaffolds (Fig. 2g). Both cells’ growth on ABM alone was shown in Fig. 2f and h. After 6 weeks in culture (n = 3) in basal media, Live/dead labelling and confocal images showed that extensive HDPSCs on both ABM-P-15 and ABM scaffolds after 6 weeks of culture (Fig. 2i and j). For both scaffold types, HDPSCs were seen to be spread across scaffold particles to form cell bridges. The clustering of the scaffolds particles was observed in the case of the ABM-P-15 scaffolds (Fig. 2i) in comparison with the same cells on ABM scaffolds (Fig. 2j). However, there was much less HBMSCs growth on both scaffold types compared to the HDPSCs. Similarly, it can be seen that P-15 enhanced the growth of HBMSCs on ABM-P-15 (Fig. 2k) in comparison to the same cells on the ABM alone scaffolds (Fig. 2l).

### SEM imaging to show cells growth and matrix deposition on ABM-P-15 and ABM scaffolds

**Fig. 3:**
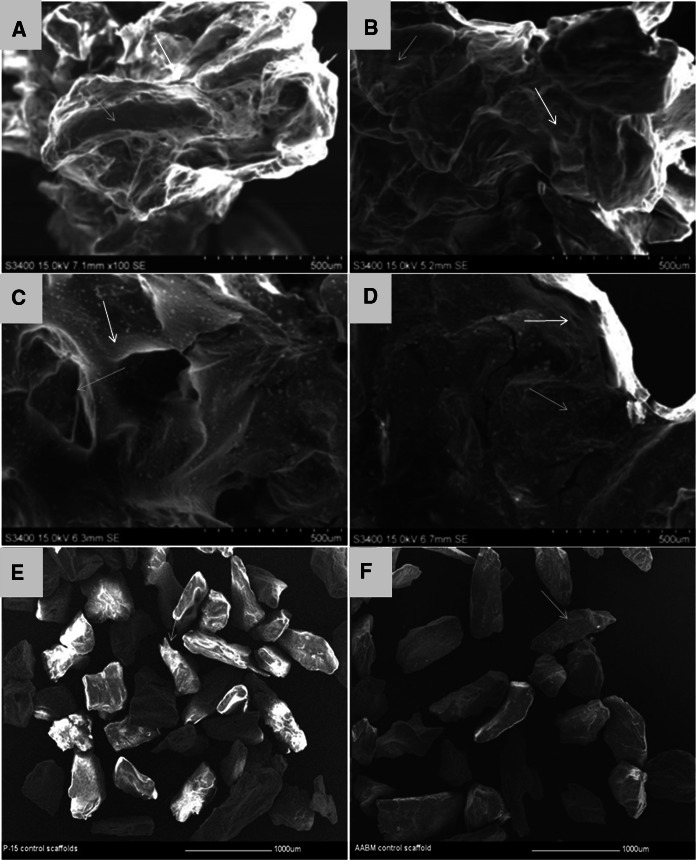
Scanning electron microscopy images of HDPSCs (**a**, **b**) and HBMSCs (**c**, **b**) after 6 weeks of in vitro culture on ABM-P-15 (**a**, **c**) and ABM (**b**, **d**), as well as both scaffolds without cells (**e**, **f**). HDPSCs and HBMSCs on both ABM -P-15 and ABM scaffolds were observed to deposit matrix (red arrows) around the scaffolds particles (blue arrows)

After 6 weeks of culture in basal medium, Scanning electron micrographs showed that HDPSCs and HBMSCs had formed clusters, presumably related to cell bridging and matrix deposition on ABM-P-15 (Fig. 3a and c) and ABM scaffolds (Fig. 3b and d). The cells on the scaffolds appeared to have formed a thick sheet-like layer encasing the scaffold particles. This was observed for both cell types and for ABM-P-15 and ABM alone scaffolds, respectively. Figure 3e and f showed the ABM-P-15 and ABM scaffolds without cells.

### ALP staining and ALP Specific activity of HDPSCs and HBMSCs on ABM-P-15 and/or ABM particles

**Fig. 4:**
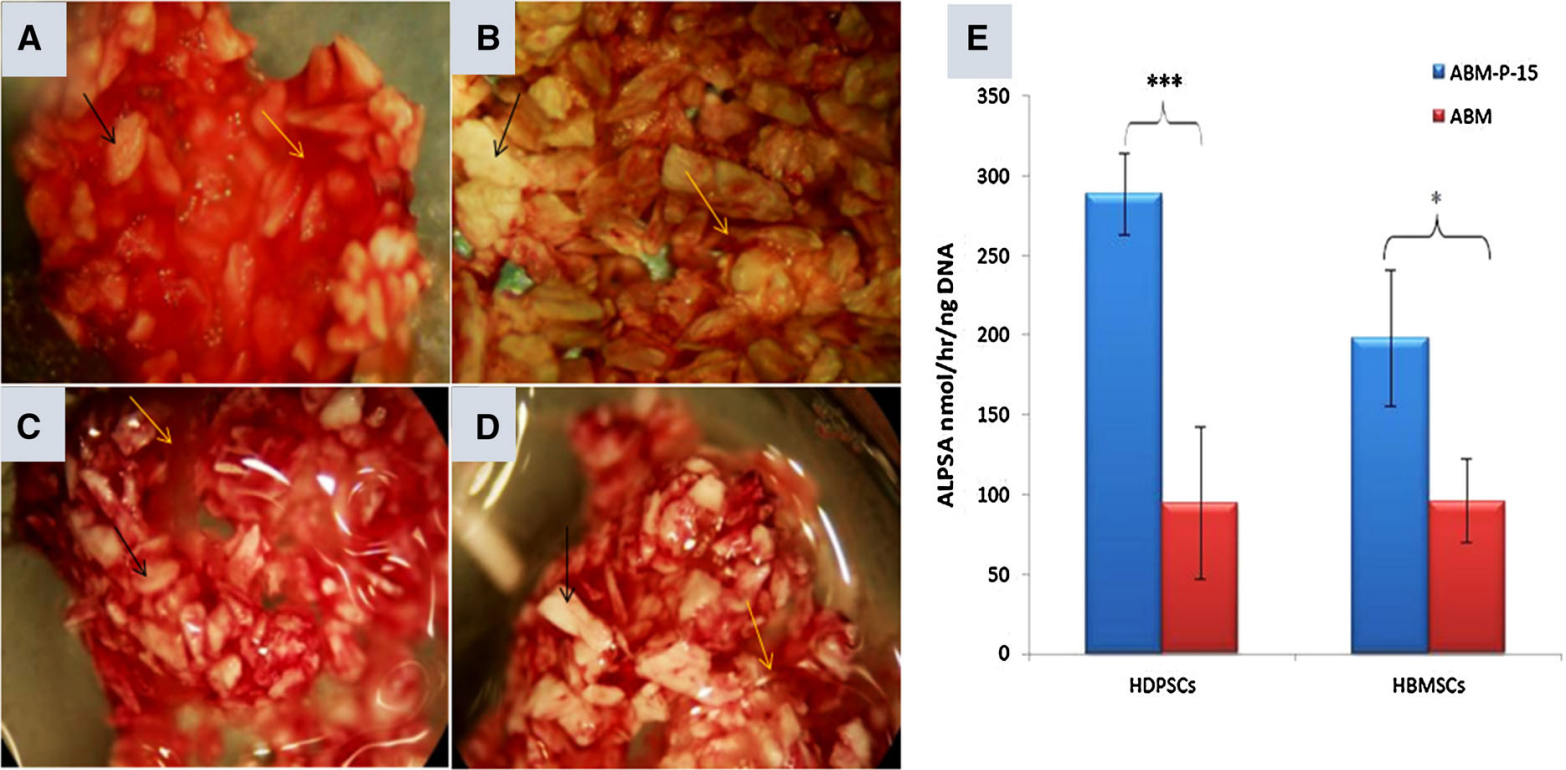
ALP staining (**a**–**d**) and quantification of ALP specific activities (**e**) for HDPSCs (**a**, **b**) and HBMSCs (**c**, **d**) after 6 weeks of culture on ABM-P-15 (**a**, **c**) and ABM (**b**, **d**) scaffolds (n = 3). *P < 0.05; ***P < 0.001

After 6 weeks of culture in basal medium, ABM-P-15 groups for both HDPSCs and HBMSCs showed enhanced ALP staining compared to that of the same cell types on ABM scaffolds (Fig. 4). There was no visible difference between HDPSCs (Fig. 4a) and HBMSCs groups on the ABM-P-15 scaffolds (Fig. 4c). For the ABM alone groups, the HBMSCs group (Fig. 4d) showed stronger ALP stain than that of the HDPSCs group (Fig. 4b). However, these were not a significant difference in the ALPSA (P > 0.05). Biochemical quantitative assays confirmed that HDPSCs cultured on ABM-P-15 scaffolds had the highest ALPSA compared to HDPSCs on ABM alone scaffolds (200% increase) (P < 0.001, Fig. 4e). Similarly, HBMSCs cultured on ABM-P-15 scaffolds also had significantly higher ALPSA compared to the cells cultured on ABM scaffolds alone (100% increase) (P < 0.05, Fig. 4e). The mean of ALPSA of HDPSCs was 30% higher than that of HBMSCs on the ABM-P-15 group. However, there was no statistic difference in the ALPSA between the two cell types (P > 0.05).

### Sirius red staining and Birefringence images to show the fibrous collagen matrix present in HDPSC-ABM-P-15 and/or ABM scaffold construct in vivo

**Fig. 5:**
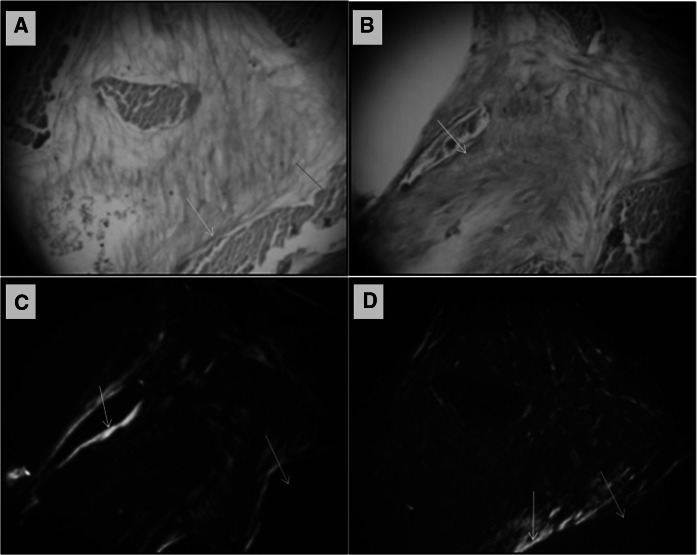
Alcian blue/Sirius red staining (**a**, **b**) and birefringence (**c**, **d**) of the fibrous collagenous matrix present in HDPSC-ABM-P-15 (**a**, **c**) and/or HDPSC-ABM (**b**, **d**) scaffold constructs after 8 weeks of in vivo implantation in a diffusion chamber model. Yellow arrows: collagen matrix formation (red or bright colour) and orientation; Blue arrows: ABM particles. Magnifications: ×200

After 8 weeks of in vivo implantation (n = 4), three out of four HDPSCs-ABM-P-15 constructs showed positive staining for Sirius red (Fig. 5a). In comparison, only one out of four HDPSCs-ABM constructs showed positive stinging for Sirius red (Fig. 5b). In the negative control groups, ABM-P-15 and ABM scaffolds without cells, there was no indication of the presence of cells or tissues within the constructs. Under polarized light microscopy, the Sirius red-stained collagen matrix exhibited birefringence, and the fibres appeared green/red/orange in colour (Fig. 5c and d). A denser and more highly organised collagen matrix was observed in the HDPSCs-ABM-P-15 constructs (Fig. 5c) compared to that in the HDPSCs-ABM constructs (Fig. 5d).

### Immuno fluorescent characterisation of the extracellular matrix of HDPSCs-ABM-P-15 and/or HDPSCs-ABM scaffolds constructs in vivo

**Fig. 6:**
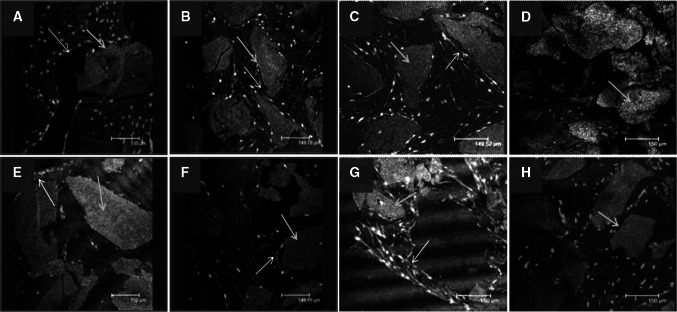
Immunofluorescent staining for type I collagen (**a**, **e**), osteocalcin (**b**, **f**), and OPN (**c**, **g**) for HDPSCs on ABM-P-15 (**a**, **b**, **c**) and ABM (**e**, **f**, **g**) scaffold constructs after 8 weeks of in vivo implantation in a diffusion chamber model. **d**, **h** The negative controls (without primary antibodies). Green staining indicates positive immunofluorescent staining (Red arrows); The blue staining indicates nuclei of cells, and grey staining (blue arrows) indicate the ABM particles

After 8 weeks of in vivo implantation, immunofluorescent staining showed that HDPSCs-ABM-P-15 groups appeared to have more and stronger positive stains (green colour, red arrows) for COL1 and OCN within the cells and extracellular matrixes, the collagen matrixes were dense and organised around individual ABM-P-15 scaffold (Fig. 6a and b) compared to that of ABM alone group (Fig. 6e and f) respectively, in which the matrixes were less organised between the scaffold particles while the most of organised matrixes were observed around the peripheral layer. In comparison, there were less staining for OCN than COL1 within the same group. The nuclei were stained as blue colour, and the ABM particles were shown as the grey colour (blue arrows). The HDPSCs on both ABM-P-15 (Fig. 6c) and ABM scaffold groups showed strong positive stains for OPN (Fig. 6g). There was no clear difference between the two groups. There were not positive stains in the negative control groups (without primary antibodies) on ABM-P-15 (Fig. 6d) and ABM alone (Fig. 6h).

## Discussion

Translational research on bone tissue engineering aims to develop cell-based bone graft material that could be employed as a substitute for the traditional grafts for bone augmentation. However, one of the current challenges in this field is the identification of an ideal combination of stem cells, scaffold material and growth factors that could be used for faster repair/regeneration of damaged bone (Panetta et al. 2009) and/or improve the implant-bone osseointegration (Jayesh and Dhinakarsamy 2015; Ting et al. 2016). In this study, the effect of ABM-P-15 on HDPSCs osteogenesis was investigated both in vitro and in vivo compared with HBMSCs with the aims of developing novel stem cell-biomaterials combinations for enhancing bone tissue repair/regeneration efficacy and improve the implant-bone interface for clinical application.

Although HBMSCs has been considered as one of the most popular stem cell sources (Squillaro et al. 2016; Yoshii et al. 2009), however, due to the limitation of getting a good quality of HBMSC and considerable very long doubling time of this cell population, resulting in a slow or low efficacy bone formation procedure. In fact, for clinical therapy, the speed for bone formation may be more important than the amount of bone formation itself (e.g. taking longer time). Therefore, researchers are looking for different alternatives for bone tissue regeneration. A number of studies showed that HDPSCs from dental pulp tissue is highly proliferative, short doubling time, multipotency, in particular with high osteogenic potential, which makes these cells alternative candidates for bone tissue regeneration (El-Gendy et al. 2015; Yamada et al. 2019). In this study, both HDPSCs and HBMSCs showed low adipogenic and chondrogenic potential but with some osteogenic potential in basal medium culture conditions. However, when cultured in inductive media, both HDPSCs and HBMSCs showed the enhanced capacity for their osteogenesis, adipogenesis, and chondrogenesis. HDPSCs group showed stronger stain for Alcian blue, which indicated more cartilage proteoglycans (Saha et al. 2013; Ullah et al. 2012) were formed in this group compared to that of HBMSCs group in basal. Similarly, the HDPSCs group showed stronger ALP positive staining than that of HBMSCs group in osteogenic inductive culture condition. These results were in agreement with the literature in supporting HDPSCs as an alternative stem cell source for bone tissue engineering (El-Gendy et al. 2013; El-Gendy et al. 2015).

Although in this study, the difference in the number of cells attached on both ABM scaffolds was not quantified, morphological observations appeared that P-15 increased the cell-binding after 24 h of seeding and enhanced the cell proliferation/cell bridge formations after 14 days of seeding for both HDPSCs and HBMSCs onto ABM-P-15 particles compared to ABM scaffolds alone, which was consistent with our previous study on HBMSCs (Yang et al. 2004) and the work of others on different cell populations (Bhatnagar et al. 1999b; Emecen et al. 2009; Lallier et al. 2003). Following long term culture (6 weeks in the basal medium in vitro), interestingly it was observed that extensive viable HDPSCs were growing on both ABM-P-15 and ABM alone scaffolds. In comparison, there were much fewer HBMSCs on both groups although there was a sign of more HBMSCs on the ABM-P-15 scaffolds than that on ABM alone scaffolds. These may be due to the higher proliferation rate and lower population doubling time for HDPSCs (Eslaminejad et al. 2010; Pisciotta et al. 2015) compared to that of HBMSCs. Bhatnagar et al. (Bhatnagar et al. 1999c) showed that P-15 stimulated ECM synthesis. In this study, both HDPSCs or HBMSCs cultured on ABM-P-15 appeared to deposit well organised ECM around the individual scaffold particles after 14 days of seeding, which holds the separate ABM particles together in clusters (Yang et al. 2004). In contrast, the cells on ABM alone were observed to be concentrated on individual scaffold particles and formed fewer cell bridges with the neighbouring scaffold particles. The enhanced cell bridge formation in cells cultured on ABM-P-15 might be attributed to the development of traditional force by the cells, which is important for the organisation of the matrix and tissue morphogenesis (Bhatnagar et al. 1999a; Schwartz 2010). This study, however, has not measured difference in the tractional force imparted by the cells cultured in the presence of ABM-P-15 and ABM scaffolds and also no characterisation of the deposited matrix by HDPSCs/HBMSCs on ABM-P-15 and ABM scaffolds.

P-15 functions as surrogate collagen in enhancing osteogenic differentiation of the adhered cells, by the up-regulation in growth factors expression such as bone morphogenetic proteins (BMPs)-2, 6 and 7. Enhanced expression of BMP-2, 6 and 7 are documented in influencing the cells’ osteogenic differentiation in an autocrine or paracrine manner (Bandyopadhyay et al. 2006; Li and Cao 2006; Nguyen et al. 2003) and are involved in the synthesis of collagen, OCN and other extracellular matrix proteins (Bhatnagar et al. 1999a, b; Locklin et al. 1999; Warren et al. 2001). ALP is a widely studied pre-osteoblastic marker that is expressed during the end of osteoblast proliferation (Lian and Stein 1995; Lu et al. 2014; Mendes et al. 2004). Immobilised P-15 on ABM scaffolds were observed to up-regulate the ALP expression of human dermal fibroblasts, HBMSCs and periodontal ligament fibroblasts (Qian and Bhatnagar 1996; Yang et al. 2004; Yuan et al. 2007) and this effect has been correlated with the increase in BMP-2 expression (Spinella-Jaegle et al. 2001). The up-regulation of ALP is essential for matrix mineralisation as it catalyses the hydrolysis of phosphomonoesters at alkaline pH (Bellows et al. 1991; Gillette and Nielsen-Preiss 2004). In this study, both HDPSCs and HBMSCs group showed much stronger ALP positive staining compared to the same cell types growth on the ABM scaffold alone after 6 weeks of in vitro culture in basal medium. Quantitative biochemical assays confirmed that the ALPSA of HDPSCs on ABM-P-15 group is 200% increase compared with that of the same cells on ABM along group. There were 100% increasing in ALPSA in HBMSCs cultured on ABM-P-15 group than that of the same cells on ABM alone group. The ALPSA of HDPSCs on ABM-P-15 group was higher than that of HBMSCs on ABM-P-15 group (32%), which was similar to the results of Kwon et al. (2015) (Kwon et al. 2015). These results may indicate that the response of tested HDPSCs to ABM-P-15 was more sensitive than the tested HBMSCs. However, there was no statistic difference in the ALPSA between the two cell types (P > 0.05).

The diffusion chamber model has been used for decades to test the tissue regenerative strategy (Ashton et al. 1980; Gundle et al. 1995; Howard et al. 2002; Nawata et al. 2005; Partridge et al. 2002; Yang et al. 2003, 2004). It can be implanted intraperitoneally in mouse or rat and provide a permissive physiological environment in supporting stem cell growth, function and tissue regeneration in vivo. The enclosed system allows the exchange of nutrients, oxygen and waste across the membrane filters but prevents the entry of host cells and tissue into the constructs (Horner et al. 2008; Lu et al. 2014). Previously, we have shown that ABM-P-15 promoted HBMSCs forming bone matrix after 6 weeks implantation (Yang et al. 2004). Similarly in this study, after 8 weeks in vivo implantation in MF1 Nu/Nu mice, the ABM-P-15 group showed highly organised collagen matrix formation within the diffusion chamber, which indicates that ABM-P-15 enhanced HDPSC bone formation compared to that of ABM alone group. These results were supported by enhanced immune fluorescent staining for COL-1 and OCN, in the ABM-P-15 group, confirming terminal differentiation of the HDPSCs. In comparison to the normal light microscope, the use of polarised microscopy for the identification of collagen orientation is preferred as it increases the specificity and resolution for the observation of the thin collagen fibres which are not detectable under normal microscopy (Junqueira et al. 1979; Rich and Whittaker 2005; Spiesz et al. 2011; Traini et al. 2006).

In the native microenvironment, the cells are under constant interaction with the extracellular matrix through the integrin receptors present in the cell membrane (De Franceschi et al. 2015; Schwartz 2010). Integrin receptors, not only function as cell adhesion molecules for the anchorage of the cells to the matrix but are also involved in the transmission of bidirectional signals across the cell, and the matrix thereby helps in the regulation of the cell proliferation, migration and differentiation (Carinci et al. 2004; Emsley et al. 2000; Jokinen et al. 2004). Similar to the native collagen fibre, the P-15 receptors have also been identified to interact with the α2β1 integrin receptors of the cells to enhance the attachment and differentiation in different cell types. The biomimetic scaffolds employed in this study mimics the autologous bone structure, where the surfaces of ABM particles are immobilised with P-15 peptides, which are molecules of the cell recognition sequence of the type 1 collagen (Bhatnagar et al. 1998; Murray et al. 2003; Pountos et al. 2016; Xu et al. 2000; Yu et al. 2011) and can initiate the cascade events for bone formation. A number of studies have also shown that ABM-P-15 enhances osteogenic differentiation and bone matrix formation using different cell types (Lindley et al. 2010; Matos et al. 2011; Vastardis et al. 2005; Yang et al. 2004; Yuan et al. 2007). The combination of ABM with P-15 and autologous HDPSCs is to mimic autologous bone graft.

## Conclusion

The current study provided direct evidence that HDPSCs contain multipotent stem cells that have a high proliferation rate and osteogenic potential compared to HBMSCs. ABM-P-15 promoted HDPSCs osteogenic differentiation and bone matrix formation both in vitro and in vivo, which indicated the potential of combining HDPSC and ABM-P15 for enhancing bone tissue engineering efficacy to meet the clinical reality in tackling fracture non-union, critical bone defect and/or implant loosening in orthopaedics and dentistry.

## Data Availability

Not applicable.
